# Biomechanical improvement of anterior talofibular ligament by augmentation repair of ligament advance reinforcement system: a cadaver study

**DOI:** 10.1186/s12893-023-02136-x

**Published:** 2023-10-10

**Authors:** Dulei Xiang, Wenming Jin, Han Li, Gen Zhao, Bao Li, Shuyuan Du, Xinwei Liu

**Affiliations:** Department of orthopedics, General Hospital of North Theater Command, 83 Wenhua Road, Shenyang, Liaoning China

**Keywords:** Anterior talofibular ligament, LARS ligament, Ligament augmentation repair, Broström repair, Biomechanics, Cadaver

## Abstract

**Background:**

Ankle sprain are one of the most frequent sports injuries. Some individuals will develop chronic lateral ankle instability (CLAI) after ankle sprain and suffer from recurrent ankle sprain. Current surgical treatment of CAI with anterior talofibular ligament (ATFL) rupture fails to restore the stability of the native ATFL. Ligament Advance Reinforcement System (LARS) augmentation repair of ATFL was developed to improve its primary stability after repaired.

**Methods:**

This study was performed to evaluate whether LARS augmentation repair of ATFL had similar stability as the modified Broström repair and the intact ATFL to maintain ankle construct stability. Standardized surgical techniques were performed on eighteen fresh frozen cadaver ankle specimens. The intact ATFL group has just undergone an ATFL exploratory surgery. The modified Broström procedure is based on anatomical repair of the ATFL with a 2.9 mm suture anchor, and the LARS procedure is an augmentation procedure of the ATFL using LARS ligaments based on the modified Broström procedure. A dynamic tensile test machine was used to assess load-to-failure testing in the three groups. The ultimate failure load and stiffness were calculated and reported from the load-displacement curve. A one-way analysis of variance was used to detect significant differences (p < 0.05) between the LARS augmentation repair, the modified Broström repair and the intact ATFL, followed by least significant difference (LSD) post-hoc tests.

**Results:**

The LARS augmentation repair group showed an increased in ultimate failure to load and stiffness compared to the other two groups. There were no significant differences in ultimate failure to load and stiffness between the modified Broström and the intact ATFL, the LARS ligament for ATFL augmentation allows for improved primary stability after repair and reduced stress on the repaired ATFL, which facilitates healing of the remnant ligament.

**Conclusions:**

The LARS augmentation repair of ATFL represents a stable technique that may allow for the ankle stability to be restored in patients with CAI after surgery.

## Background

Lateral ankle ligaments damage, which can be easily caused by lateral ankle sprain, can severely affect ankle function and life in athletic and physically active individuals [1, 2]. In lateral ankle ligaments, Anterior talofibular ligament (ATFL) is the weakest part and usually the first and most common injured ligament, especially only on the ATFL superior fascicle [3, 4]. Over 30% of patients with lateral ankle ligaments damage developed into chronic lateral ankle instability (CLAI) eventually after experiencing recurrent sprain of the lateral ankle [5]. CLAI causes chronic ankle pain, repeated ankle rollover and sprains, proprioceptive impairment, and degenerative changes [6–9]. These symptoms cause ankle function dysfunction and can seriously affect people’s life, requiring treatment to restore ankle function.​.

Treatment for CLAI includes conservative treatment and surgical treatment, and there is no optimal treatment.​ Although conservative treatment is initially required after ankle sprains and is effective in restoring ankle function, surgery is often performed to ameliorate ankle symptoms when a patient develops CLAI with persistent ankle pain and instability [10]. Among the lot of surgical treatments for CLAI, the Broström repair procedure is currently the gold standard surgical treatment and was first reported to repair ATFL in 1966 [11]. ​Several studies have demonstrated that the Broström repair procedure improves symptoms of abnormal ankle laxity with good clinical efficacy [12, 13], but other studies identified the Broström repair has some limitations. ​For example, Waldrop et al. [14] showed that the ATFL strength after the Broström repair with direct suture repair is lower than that ATFL fixation with suture anchor and intact ATFL. One of the major insights to emerge from this study is that ATFL cannot return to its original state regardless of the surgery used to repair it [14, 15], because early weight bearing and early rehabilitation after the Broström repair may affect the healing of the repaired ATFL and cause it to elongate​, and prolonged immobilization and bracing after Broström repair can cause ankle stiffness and associated muscle atrophy [16, 17]. As a result, to restore ankle function as much as possible after CLAI, the ideal treatment requires the ability to perform rehabilitation training while protecting the repaired ATFL.​[16, 18, 19].

Ligament advanced reinforcement system (LARS) has been proposed and widely used for anterior cruciate ligament (ACL) reconstruction to restore mechanical and anatomical properties of ACL [20, 21]. Extensive literature indicated good clinic efficacy and superiority of ACL reconstruction with LARS ligaments for high fatigue resistance, and biopsies have additionally shown complete cellular and connective tissue ingrowth [22, 23]. LARS was also used for ATFL reconstruction in CLAI patients, which got excellent clinic efficacy and achieved good ankle stability compared to the modified Broström repair [24, 25]. However, some studies demonstrated that limitations of ATFL reconstruction in ankle activity following and the removal of ATFL remnants can potentially affect ankle functional recovery similar to remnant-preserved anterior cruciate ligament (ACL) reconstruction [26, 27], and ATFL remnant preservation was benefit for proprioceptive recovery [28]. In addition, early range of motion rehabilitation has also been demonstrated to improve ankle strength, mechanical stability, and return to activity outcomes compared to cast immobilization [29, 30].

​ These studies suggest that the treatment with ATFL remnant protection and allowing for early rehabilitation may be a better procedure for CLAI patients. Our team designed all arthroscopic ATFL augmentation repair procedure by using the LARS ligament as the supporting structure to enhance the primary stability of the repaired ATFL. The objective of this study was to introduce the LARS augmentation repair procedure for CLAI treatment, and the biomechanical properties of ATFL after LARS augmentation repair and modified Broström repair were investigated for the ultimate failure load and stiffness compared to the intact ATFL in cadaver specimens. ​We hypothesized that ATFL augmentation repair procedures may provide improved mechanical strength and enhanced ankle stability. Further, the findings of this study can provide a theoretical foundation for clinically reasonable treatments.

## Methods

### Study design

18 fresh-frozen adult cadaveric ankles (mean age: 57.1 years; range, 34 to 65 years) were utilized in this study. These specimens underwent different surgical procedures for restore ATFL before biomechanical test of ultimate failure load and stiffness of repaired ATFL. ​All specimens were thawed, dissected, and examined, and there was no ligament ruptures or ankle surgeries and a history of cancer was not listed as a cause of death. An anterior drawer test was performed to assess the ligament, which was not ruptured. Specifically, the lower limb is steadied by one hand, the heel is firmly grasped by the other hand, and the heel is then moved, the calcaneus not being translated anteriorly with a solid terminal feel. Specimens were randomly assigned to three groups: (1) intact ATFL, (2) modified Broström repair of ATFL, and (3) LARS augmentation repair of ATFL. All specimens were kept at -20℃ and thawed at room temperature for 24 h before experiment, and were kept moist with saline to prevent tissue desiccation throughout biomechanical testing.

### Procedures

#### Surgical approach

**Fig. 1:**
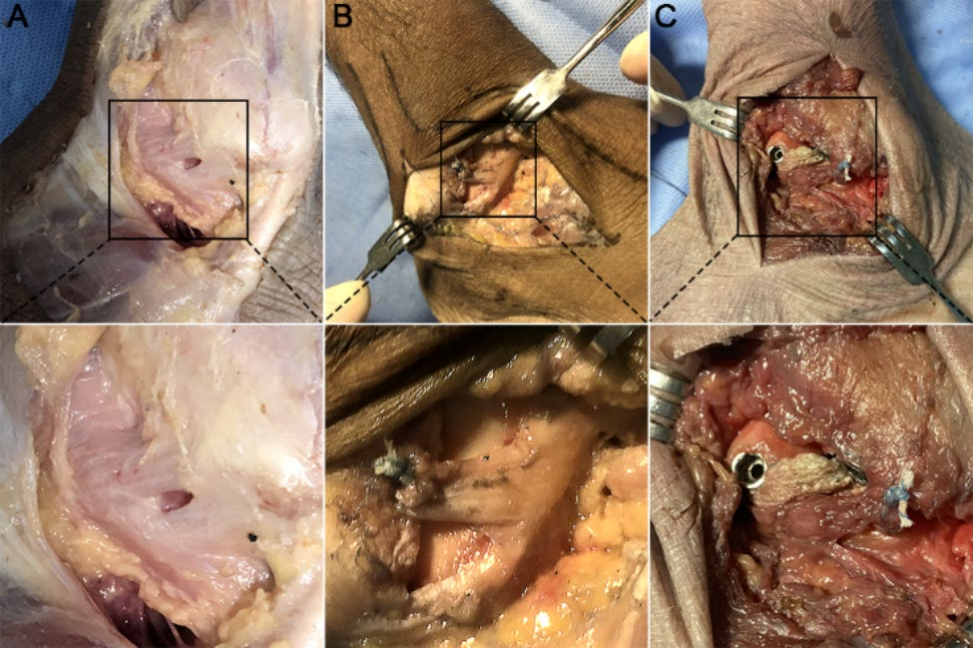
(**A**) Intact ATFL ligament (**B**) All arthroscopic Modified Broström repair of ATFL (C) All arthroscopic LARS augmentation repair of ATFL. Modified Broström repair with one 2.9-mm suture anchor. Augmentation with one 2.9-mm suture anchor, two 4.7 mm interference screws and one Ligament Advance Reinforcement System

All surgical procedures were performed in accordance with the standard protocols (Fig. 1). A J-shaped incision was performed anteriorly from the distal tip of fibula along its proximal anterior boundary to the level of the ankle mortise. The subcutaneous tissues were separated, and the joint capsule was opened, allowing exposure of the ATFL, the calcaneofibular ligament (CFL) and their attachment points on fibula and talus. The ATFL were inspected for prior injury, and the procedure for intact ATFL group was stopped after this step. Subsequently, the ATFL was cut off along the distal fibula in other groups. The laxity of the ankle was assessed by using the non-instrumental anterior drawer test.

Modified Broström Repair of ATFL was performed on each specimen in Group2. The surgical repair consists of one 2.9-mm suture anchor (Smith & Nephew, America). An anchor was placed in the center of the ATFL footprint on fibula and the sutures were tied over the top of the ATFL footprint to simulate the arthroscopic technique of ATFL repair. An anterior drawer test was subsequently performed to check the stability of the ankle.

​ LARS augmentation repair for ATFL were performed on each specimen in Group3. ​Briefly, LARS augmentation repair is performed by adding the augmentation step with LARS to the Brostrom repair. The surgical augmentation repair consists of one LARS ligament (LARS, France), two 4.7 mm interference screws (LARS, France) and one 2.9-mm suture anchor (Smith & Nephew, America). Two 3.5 mm bone tunnels were drilled into the fibula and talus, located proximal to the ATFL footprint on the fibula and talus and close to the footprint. The modified Broström repair protocol is initiated after the bone tunnel is established. The LARS ligament was folded and cut to a length of 30 mm and marked on 10 mm apart at the each edge. The LARS ligament was passed into the tunnel and fixed with proper tension on the fibula and talus. An anterior drawer test was subsequently performed to check the stability of the ankle.

#### Specimen Preparation

Similar methods of ankles preparation and fixation before biomechanical testing have been published previously, and new data and modifications to the original model are described in detail here [31, 32]. ​ATFL was isolated in all specimens to reduce the effect of other tissues, following the standard protocol prior to biomechanical testing. ​The tibia, original soft tissues, and muscle attachments on the fibula were resected, except for the ATFL footprints on the distal fibula and lateral talus, leaving the foot structure and skin intact. ​Only the intact ATFL, the repaired ATFL, and the LARS ligament augmented ATFL were retained for the next biomechanical test, and the other ligaments were cut.

**Fig. 2:**
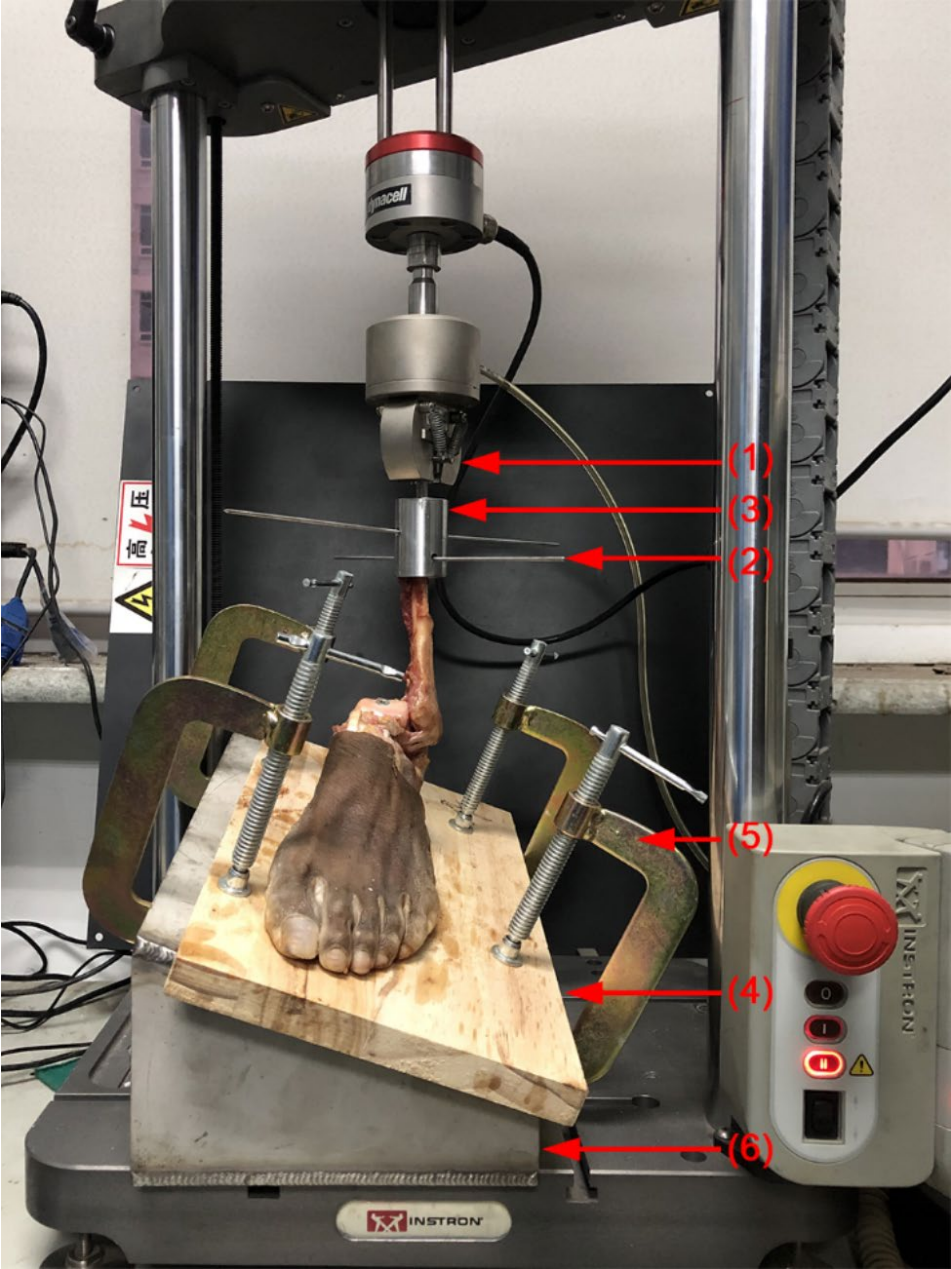
Biomechanical test set-up. The left ankle specimen was (1) mounted to the test machine (2) using a custom steel cup. (3) Kirschner wires were used to prevent the fibula movement. (4)The foot was fixed to a wood plate, (5) which was mounted to the custom holder by clamps. (6) The custom holder secured to the machine mimicked ankles sprain to perform the position of maximum tension of ATFL.

To eliminate the displacement error, all ankles was fixation on plate before biomechanical testing. Each specimen was rigidly fixed on a plate with five screws (6 mm diameters) which were used to fix the dorsum of feet, calcaneus and subtalar joint. The wood plate was mounted with a custom holder to simulate the ankle sprain position at 20 degrees of inversion and 10 degrees of plantar flexion. Furthermore, the fibula was secured in a custom cup by two Kirschner wires to ensure that the fibula remains perpendicular to the floor during the machine loading. The holder was secured to the test machine (Fig. 2). Mechanical tensile-stress experiments were performed by using a dynamic tensile testing machine (Instron E1000, Norwood, MA, America).

### Biomechanical testing

At the start of the biomechanical test, each Specimen was preloaded to 5 N for slack removal, and gradually loaded to 15 N over 10 s. The force was held constant for a period of 5 s to remove potential creep deformations. Subsequently, the specimen was loaded to failure by displacing the fibula at a rate of 20 mm/min. The data of time, force and displacement of fibula were recorded by Instron BlueHill 2 software (Instron Corporation, Norwood, America). The failure modes of the specimens were recorded. Further calculation and statistical analysis were performed with Excel (Microsoft Inc, Seattle, America). The ultimate failure (N) load was recorded from the load-displacement curve, and the stiffness (N/mm) was calculated from the slope of the curve.

### Statistical analysis

The statistical analysis was performed using SPSS Statistics version 23 (IBM, America). Data among groups were compared with one-way analysis of variance (one-way ANOVA) followed by least significant difference (LSD) post-hoc tests. The ANOVA was used to determine whether there are any statistically significant differences between the means of ultimate failure load and stiffness of the different groups, and the statistically significant difference was determined at p < 0.05.

## Results

​ There were no significant between-group differences in each group in any demographic variable (Table 1). The outcome of ultimate failure load, stiffness, and mechanism of failure of treatment groups were compared to the intact ATFL, and then the outcome of treatment groups were compared to each other (Table 2).

**Table 1:** Demographics the certain specimens of the different groups

Group	Age mean (range), y	Male/female (n)	Right/left (n)
Intact	53.8 (34–62)	4:2	4:2
All arthroscopic Modified Broström	57.5 (50–65)	3:3	2:4
All arthroscopic LARS augmentation	60.0 (52–64)	5:1	2:4
All specimens	57.1 (34–65)	12:6	8:10

**Table 2:** Mean ultimate failure load and Stiffness of the different groups compared to the intact ATFL

	Ultimate failure load			Stiffness	
Group	Mean ± SD, N	P Value		Mean ± SD, N/mm	P Value
Intact	160.9 ± 54.6	-		14.7 ± 5.6	-
All arthroscopic Modified Broström	194.1 ± 61.2	0.376		17.4 ± 9.3	0.571
All arthroscopic LARS augmentation	356.3 ± 72.1	0.000		29.1 ± 8.6	0.007

**Fig. 3:**
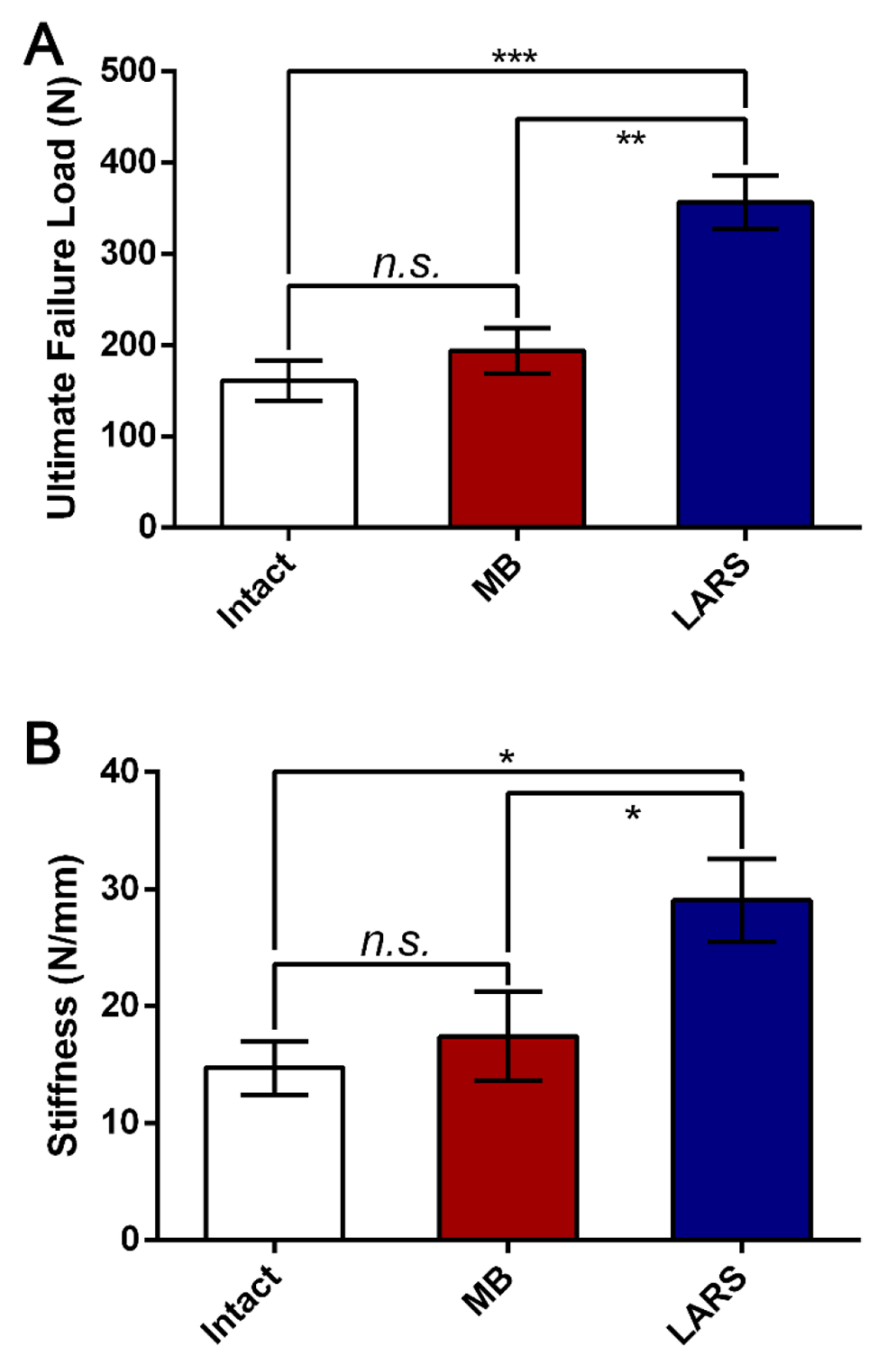
**A, B** Ultimate failure load and Stiffness for different groups of ATFL

The mean ultimate failure load was significantly higher in LARS augmentation repair group (356.3 ± 72.1 N) than in the intact ATFL group (160.9 ± 54.6 N) (P = 0.000), and the mean ultimate failure load was significantly higher in LARS augmentation repair group than in modified Broström repair group (194.1 ± 61.2 N) (P = 0.000). The mean ultimate failure load was no significant difference between modified Broström repair group and intact ATFL group (P = 0.376) (Fig. 3).

The mean stiffness was significantly higher in LARS augmentation repair group (29.1 ± 8.6 N/mm) than in the intact ATFL group (14.7 ± 5.6 N/mm) (P = 0.007), and the mean stiffness was also significantly higher in LARS augmentation repair than in modified Broström repair group (17.4 ± 9.3 N/mm) (P = 0.024). The mean stiffness was no significant difference between modified Broström repair group and intact ATFL group (P = 0.571) (Fig. 3). The LARS augmentation appears to protect against the major failure mode of ligament-suture interface rupture in the Broström repair with suture anchor.

## Discussion

In this study, we examined biomechanical property of ATFL of the ultimate failure load and stiffness after different surgical treatment of CLAI. Our results indicate that LARS augmentation repair of ATFL lead to better biomechanical results than modified Broström repair and intact ATFL. The biomechanical properties of intact ATFL provide a further reinforcement of prior findings. In the results, the group2 dose was able to withstand physiological stress, but this was a lab-based study, and the stress on the ligaments in the fresh-frozen cadaver was probably lower than in a live human. The true ligament stress is probably much higher. While the dominant failure mode of group2 is ligament-suture interface rupture, the ligament-suture interface stress settles and does not change with material.

The results of biomechanical property of repaired ATFL in present study are in accordance with the other studies. Our study also showed that the ultimate failure load and stiffness were significantly higher in LARS augmentation repair group than in the other two groups in the biomechanical test on cadaver models. The mean ultimate failure load and stiffness were approximately 50% higher in LARS augmentation repair procedure compared to the intact ATFL, and that the date were significantly different in the two surgical treatments. Data of the mean ultimate failure load of the intact ATFL (160.9 ± 54.6 N) in present study closely resembles the results reported by Attarian et al. [33] (138.9 N ± 23.5 N). The modified Broström repair has been widely used over last decades and is considered as the ‘gold standard’ surgical treatment for CALI patients [34]. Autografts or allografts for ATFL reconstruction in CLAI are commonly used in cases where the quality of the ligament tissue is poor and the remnant is not suitable for repair [35]. However, ligament reconstruction procedures may not have a significant advantage over anatomical repair procedures in previous biomechanical studies. ​High medical cost burden and allograft rejection remain concerns for reconstruction treatment [36, 37]. The procedure in the present study combined the Broström repair for in-situ ATFL repair with an additional LARS artificial ligament. In-situ ligament repair can maintain the histological and immunohistochemical signatures of the neural receptors responsible for proprioception. Moreover, rehabilitation programs based on proprioception are becoming more popular in patients with joint injuries [28]. While early training in range of motion after ligament repair is beneficial for effective rehabilitation, especially in proprioception recovery, several studies have emphasized the importance of protection from excessive stress during the early post-operative rehabilitation phase after Broström repair [14, 28]. Lengthening of 20% in the ATFL after Broström repair with unprotected mobilization [38]. The elongation of ligaments during early mobilization has been demonstrated in biomechanical studies, suggesting the need for an additional device that provides great initial stability and allows for accelerated rehabilitation [14, 17].The present study provides novel and important biomechanical information on procedures of LARS augmentation repair of ATFL and modified Broström repair of ATFL. We demonstrate that LARS augmentation repair procedure can provide enough strength to resist stretching of repaired ATFL and the excellent biomechanical property of LARS augmentation repair allow implementation of early rehabilitation to get better recovery of ankle function.

In the present study, a novel procedure, LARS augmentation repair of ATFL, was performed to address the above issues. ATFL augmentation repair was designed for LARS ligament implantation close to the ATFL footprint. We only repaired the ATFL in the present study, which can provide sufficient strength for lateral ankle stability. The previous study proved that only repair ATFL resulted in similar outcomes to repair ATFL and calcaneofibular ligaments (CFL) [39].The LARS ligament, as a strong synthetic material, has been shown to replicate the strength and stiffness of native ligaments and has been available for decades as a treatment for knee cruciate ligament injuries [21]. Several studies have suggested that aggressive rehabilitation and rapid return to sport can be achieved after the LARS reconstruction of an ACL injured [20]. LARS ligament can overcome the issues of rejection, graft failure and the risks of synovitis that occur in Autografts or allografts used for reconstruction. LARS consists of multiple parallel longitudinal fibers that provide a biological scaffold for mimicking native ligaments. Fibroblastic implantation between the fibers acts as a viscoelastic element for ligament protection against friction at the ligament and bone tunnel junctions [22]. Moreover, the LARS ligament, which serves as a secondary stabilizer for ankle stability, was used in this procedure for providing extra great strength to the native ATFL. In this way, the LARS augmentation structure not only facilitates the healing process of the native ATFL, but also allows for accelerated rehabilitation following surgery. In the present study, none of the LARS augmentation repair group failed due to interference screws pull-out. The failure of the modified Broström repair group mostly occurred at the ligament-suture interface, suggesting that initial augmentation may be effective in avoiding such a failure after the modified Broström repair.

There are several limitations associated with this biomechanical study. Firstly, the dates in the present study should be used with caution when comparing with other biomechanical studies of CLAI treatment, given that no similar LARS procedure has been identified, differences in ankle custom fixtures and measurement methods. ​And this study is a lab-based study, where the ligaments are not actually torn, stretched. Secondly, the number of ankle specimens in the current study was relatively small. Nonetheless, this is a common problem in all biomechanical studies of ankle lateral ligament repair. Besides, the average age in this study was relatively high (68 years), which might not reflect the majority of patients with CLAI and the bone quality was weaker in elderly patients than in younger patients. The mean age of the specimens is similar to those reported elsewhere in the literature. Thirdly, the failure modes of group2 and proup3 are not actually appear to ligament torn and stretched, and most of the failure happened to the ligament-suture interface and ligament-screw interface. ​Fourthly, the LARS augmentation procedure requires more surgical time and additional LARS ligament costs compared to the modified Broström repair. And the biologic healing effect of the ATFL on patients could not be assessed and the results in the present study only represent the initial state after surgery. Finally, and clinical efficacy of LARS augmentation treatment in CLAI patients remains unclear based on available data. Additional research is necessary to assess clinical benefit with long-term follow-up data, and we will be pursuing this new procedure in the clinic to investigate its clinical efficacy in the near future.

## Conclusions

In summary, our results suggest that LARS augmentation repair improves the biomechanical property of ATFL with suture anchor repair in the fresh-frozen cadaver specimens. This study is pioneer research, the remnant of ATFL was repaired and LARS provide an extra strength for initial ankle stability. Accelerated rehabilitation may be used to get better recovery of ankle function with LARS protection of repaired ATFL.

## Data Availability

The data used to support the findings of this study are available from the corresponding author upon request.
